# Hunting and Outdoor Recreation Affect Large Herbivore Activity Patterns More Than Natural Predators in a Human‐Dominated Landscape

**DOI:** 10.1002/ece3.73033

**Published:** 2026-02-15

**Authors:** Martín Boer‐Cueva, Giulia Bombieri, Emma Centomo, Piergiovanni Partel, Enrico Dorigatti, Enrico Ferraro, Ilaria Greco, Francesco Rovero, Marco Salvatori

**Affiliations:** ^1^ Department of Ecology, Faculty of Sciences Autonomous University of Madrid (UAM) Madrid Spain; ^2^ Department of Biodiversity, Ecology and Evolution, Faculty of Biological Sciences Complutense University of Madrid Madrid Spain; ^3^ Conservation Biology Unit, Research and Museum Collections Office MUSE—Science Museum Trento Italy; ^4^ Nature Park Paneveggio Pale di San Martino Primiero San Martino di Castrozza Italy; ^5^ ACT—Trentino Hunting Association APS Trento Italy; ^6^ Department of Biology University of Firenze Sesto Fiorentino Italy

**Keywords:** human disturbance, human–wildlife coexistence, hunting, outdoor recreation, predator–prey interactions, red deer, wolf

## Abstract

Across Europe, landscapes where large carnivores, large herbivores and human communities coexist are expanding, reflecting the widespread recovery of large mammal populations in recent decades. The influence of top‐down effects of wolves on large herbivores has been extensively studied in areas with relatively little anthropogenic disturbance, but less is known about their effect in human‐dominated landscapes. We systematically collected camera‐trap data over five consecutive autumn hunting seasons in an area of the eastern Alps which is intensely frequented by tourists and trekkers, and partially open to ungulate hunting. We used a quasi‐experimental design, with half of the sampling sites located within nonhunting areas and half outside. Applying generalised additive mixed models (GAMMs) with cyclic cubic splines we investigated the effect of wolf, as well as lethal (hunting) and nonlethal (recreational) human activities on red deer spatiotemporal activity pattern. Similarly, we analysed the effect of recreational activities and red deer site‐use on the spatiotemporal activity pattern of wolves. Hunting was associated with overall lower red deer activity, as well as reduced dawn–dusk peaks and diurnality. Crucially, hunting interacted with outdoor recreation exacerbating its impact, with major changes to red deer activity curve. Wolf site‐use did not have a significant effect on the shape of red deer temporal curve. Wolves were markedly more active in areas highly used by red deer, and remained strongly nocturnal even where human activity was scarce. Our results show that humans, through both lethal and nonlethal activities, elicit stronger responses in red deer than their natural predator. Behavioural constraints imposed by humans on red deer, coupled with the cursorial predatory strategy of wolves, likely limit the possibility of wolf avoidance by red deer. In human‐dominated European landscapes, human disturbance can therefore override natural predator–prey dynamics, reshaping behavioural landscapes and potentially increasing predator and prey spatiotemporal co‐occurrence.

## Graphical abstract

Through a quasi‐experimental design including 21 hunted and 21 control sites sampled across 5 years, combined with an integrated spatiotemporal statistical approach, we found that hunting and outdoor recreation were associated with overall lower red deer activity, as well as reduced dawn–dusk peaks and increased nocturnality, respectively. Interestingly, wolf site use did not have a significant effect on the shape of red deer activity curve while hunting interacted with outdoor recreation exacerbating its effect (stronger avoidance of recreation in presence of hunting). Wolves were markedly more active in areas highly used by red deer, and remained strongly nocturnal even where human activity was scarce.

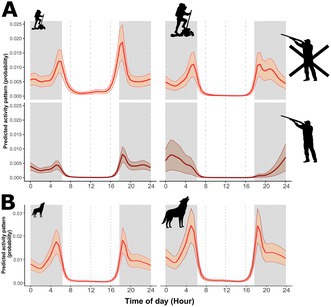

## 1 Introduction

Despite the high human population density and the pervasive presence of infrastructure and urban areas, European landscapes have recently witnessed an increase in population size and range of many large mammals (Passoni et al. 2024). Rural land abandonment and more sustainable harvesting have allowed the recovery of large herbivores, which in turn have facilitated the return of large carnivores (Linnell et al. 2020). Taking further advantage from legal protection, large predators have recently regained part of their former ranges, re‐establishing predator–prey relationships after centuries of absence in many European areas (Chapron et al. 2014). Although the effects of large carnivores on their prey have been widely studied in relatively undisturbed ecosystems in North America (Gompper 2002; Hebblewhite et al. 2005; Levi and Wilmers 2012), their relative importance compared to human influences—such as recreational hunting and outdoor activities—remains poorly understood in Europe's human‐dominated landscapes (Gerber et al. 2024). In these systems, high levels of anthropogenic disturbance coexist with both large herbivores and their predators, and emerging evidence suggests that human pressures may modulate or even weaken the ecological effects of large carnivores (Kuijper et al. 2016; van Beeck Calkoen et al. 2023).

Wildlife responses to human activities are highly context‐dependent and can vary along a gradient from avoidance to tolerance, up to attraction (Samia et al. 2015; Zenth et al. 2025). Factors such as anthropogenic food provisioning, historical human persecution, exposure to nonlethal human activities and hunting ultimately shape behavioural responses of wildlife to humans (Zenth et al. 2025).

According to the human ‘super‐predator’ hypothesis (Smith et al. 2017), humans can be exceptionally lethal for wildlife via hunting, driving profound behavioural shifts across multiple trophic levels (Darimont et al. 2015; Clinchy et al. 2016; Crawford et al. 2022; Zanette et al. 2023). The pressure of hunting activities has been shown to outweigh that of natural predators in shaping large herbivores' behaviour (Ciuti et al. 2012), evoking proactive and reactive antipredator responses in game species (Ikeda and Koizumi 2024; van Roekel et al. 2024; Vanderlocht, Robira, et al. 2025). For instance, red deer ( *Cervus elaphus* ) appear to proactively avoid humans by shifting habitat selection to safer areas with dense forest during the hunting season and increase selection of risky habitats during nighttime when hunting does not occur (Vanderlocht, Robira, et al. 2025).

Also, nonlethal anthropogenic stimuli can be perceived by wildlife as danger cues and—according to the ‘risk‐disturbance hypothesis’—trigger safety‐foraging trade‐offs similar to natural predation (Frid and Dill 2002; Lima and Bednekoff 1999). Outdoor recreation, for example, can induce spatiotemporal avoidance behaviours in large mammals, a phenomenon of increasing conservation management relevance as nature‐based tourism is rapidly gaining popularity worldwide (Balmford et al. 2015; Larson et al. 2016; Salvatori et al. 2024). Exposure and connection with nature have a proven positive effect on human wellbeing (Capaldi et al. 2015), and while they can foster positive attitudes towards conservation (Richardson et al. 2020), they can also generate adverse ecological effects, modifying wildlife assemblages, species distribution and behaviour (Reed and Merenlender 2008; Sytsma et al. 2022). Several studies reported a negative relationship between ungulates and human recreation; for example, in the USA, elk ( *Cervus canadensis* ) and moose ( *Alces alces* ) both decreased their site use intensity or feeding time in areas with higher human presence (Ciuti et al. 2012; Anderson et al. 2023). These human‐avoidance behaviours essentially decrease the amount of available suitable habitat (Gaynor et al. 2025; Smith et al. 2024) and constrain the timing of activity (Clinchy et al. 2016; Suraci et al. 2019; Oberosler et al. 2017), despite some evidence showing considerable plasticity and resilience of large herbivores to human disturbance (Salvatori et al. 2023). Both correlation and experimental studies have demonstrated that large predators also fear humans and adopt avoidance behaviours similar to those observed in other wildlife species (Gaynor et al. 2018; Ordiz et al. 2021; Bryan et al. 2015; Smith et al. 2017; Kasper et al. 2025).

Nonlethal recreation may also combine with lethal human activities, hampering the behavioural responses of ungulates towards natural predators. These cumulative pressures can hinder temporal shifts (Bonnot et al. 2020; Vanderlocht, Donini, et al. 2025), ultimately forcing a greater spatiotemporal co‐occurrence between prey and predator (Patten et al. 2019). According to theoretical frameworks, the risk of being hunted is a key driver of how wildlife behaves during non‐lethal encounters, shifting responses along a spectrum from tolerance to avoidance (Zenth et al. 2025; Rempfler et al. 2025). For these reasons, responses to outdoor recreation might be mediated by the presence of lethal human activities, with stronger avoidance expected in the presence of hunting, and vice versa higher tolerance in hunting‐free areas. However, understanding the potential interaction between these two types of human activities remains challenging, with most studies considering them separately (e.g., Kumar et al. 2023; Liu et al. 2023).

In areas where humans hunt, and where outdoor activities and large predators co‐occur, large herbivores must cope with a multifaceted landscape of fear, with multiple and potentially contrasting predatory pressures (Bonnot et al. 2020; Lone et al. 2014). Natural predation by wolves ( *Canis lupus* ) has been shown to engender spatial avoidance of riskier areas by red deer (Kuijper et al. 2014), with less time spent foraging in areas with wolf scent marking (van Beeck Calkoen et al. 2021). More broadly, studies have also reported increases in vigilance, reduced foraging (Creel et al. 2005; Kuijper et al. 2014; Weterings et al. 2022) and temporal activity shifts: for example, Vanderlocht, Donini, et al. (2025) found that red deer had a long‐lasting increase in diurnality in response to the recolonisation by wolves. In the Alps, red deer usually constitute the main food item in wolf diet (Gazzola et al. 2005), underscoring the tight predator–prey interaction that links these two species and the potential for a landscape‐of‐wolf‐fear affecting red deer behaviour. However, Gerber et al. (2024) highlight that the influence of wolves on ungulates in human‐dominated landscapes remains poorly understood. Furthermore, in cases when prey species perceive humans as a lower threat relative to their natural predators, they may exploit human presence or periods of high human activity to reduce their risk of predation by natural predators—a concept known as the human shield hypothesis (HSH) (Berger 2007; Muhly et al. 2011; Gaynor et al. 2025). Although the HSH has been studied and analysed across many ecosystems and species, it appears to be a rare and highly case‐dependent phenomenon, often incorrectly claimed (Gaynor et al. 2025). The rise of both the human super predator and HSH in the ecological literature poses an interesting yet conceptually challenging question, as it may be difficult to predict how species respond to human presence.

**FIGURE 1:**
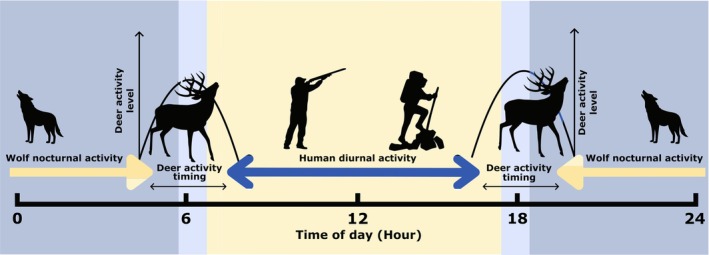
Schematic diagram of predicted effects of hunting, outdoor recreation and wolves on red deer diel activity pattern in the study area, eastern Italian Alps. Yellow arrows indicate the hypothesised effect of increased diurnality of red deer in response to wolves, while the blue arrows show the hypothesised effect of increased nocturnality of red deer in response to hunting and outdoor recreation. The net effect of these opposing drivers will depend on the relative perceived risk of humans and wolves by red deer. The shift from blue (nighttime) to yellow (daylight) background is centred on the sunrise and sunset times for early October in the study area.

In this study, we coupled systematic camera‐trapping with a quasi‐experimental design to assess the spatio‐temporal responses of red deer to hunting, outdoor recreation, and grey wolves during five consecutive years during late summer—autumn in a human‐dominated area of the eastern Alps. The season chosen corresponded to the ungulate hunting season and a period of high mobility of wolves, with cubs of the year having left the rendez‐vous sites. We hypothesised spatial avoidance by red deer of both human‐related and wolf‐related perceived risk, and thus predicted that red deer activity level (i.e., frequency of site‐use) would decrease in relation to more intense outdoor recreation (risk‐disturbance hypothesis; Prediction P1), higher wolf activity (P2) and at sites where hunting is permitted (P3). However, we hypothesised that these drivers would exert opposite pressures on deer activity timing (Figure 1): outdoor recreation and hunting would push red deer towards nocturnality (P4 and P5), whereas wolves would drive ungulate behaviour towards diurnality (P6; Figure 1). Following Zenth et al. (2025), we also predicted that responses to outdoor recreation would be mediated by hunting, with stronger avoidance within hunting areas (P7). In accordance with the human super‐predator hypothesis, we predicted that the activity level and activity pattern of wolves would also be influenced by outdoor recreation, with an expected reduction in activity level (P8) and an increase in nocturnality (P9).

## 2 Material and Methods

### 2.1 Study Area

**FIGURE 2:**
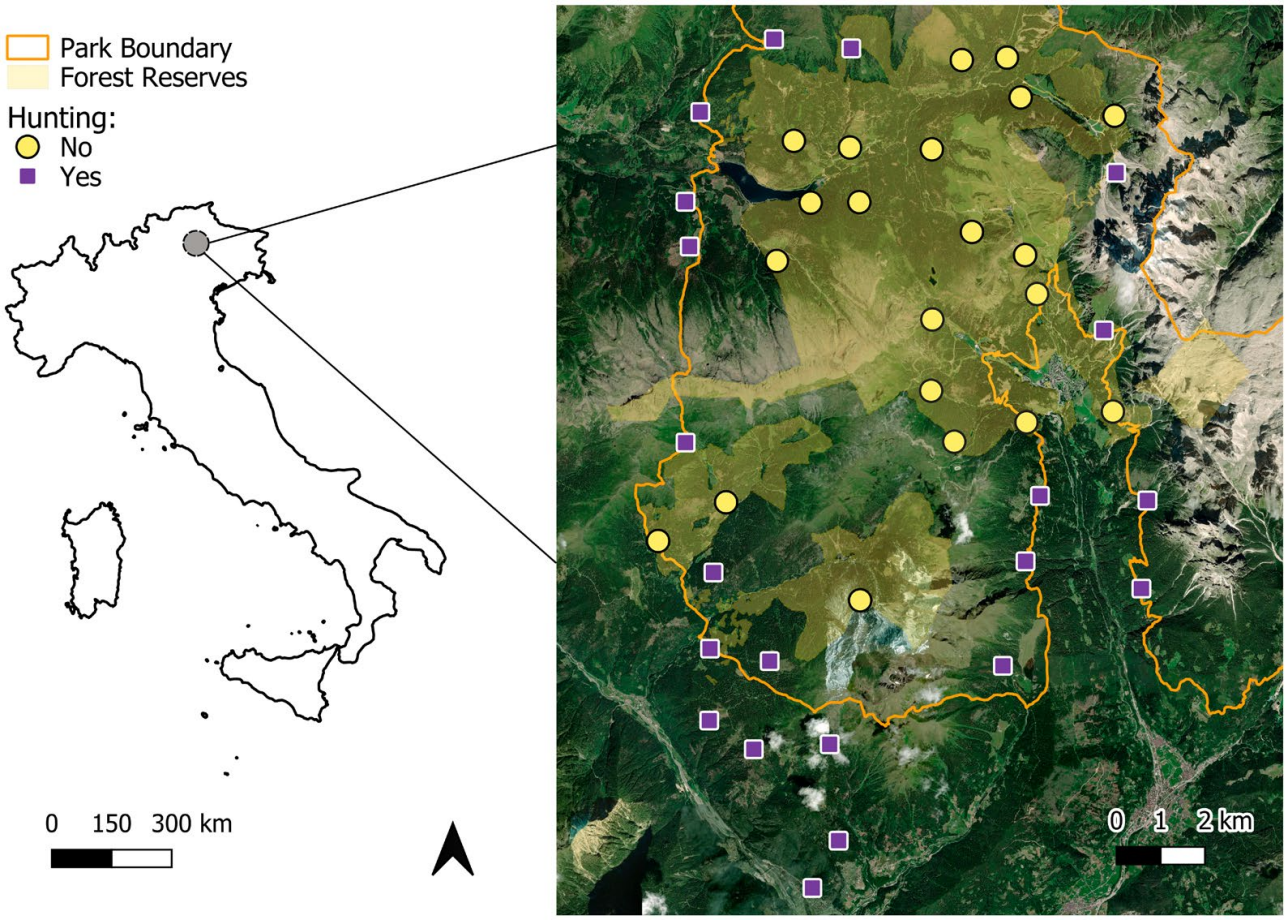
Map of the study area in the eastern Italian Alps. Purple squares and yellow circles show the locations of camera traps for sites that permit or do not permit hunting, respectively. The orange line indicates the border of the protected area Paneveggio Pale di San Martino Nature Park and the yellow polygons indicate forest reserves where hunting is forbidden. Base map satellite imagery: ESRI world imagery (ESRI Satellite https://server.arcgisonline.com/ArcGIS/rest/services/World_Imagery/MapServer/tile/%7bz%7d/%7by%7d/%7bx%7d).

Our study was conducted in the protected area of Paneveggio Pale di San Martino Nature Park and adjacent areas (46° 120 N, 11° 480 E) in the Dolomites, eastern Italian Alps (Figure 2). The park and its surroundings are characterised by being a mountainous, alpine area—with elevation ranging from approximately 600 to 3192 m a.s.l. The vegetation consists of predominantly coniferous species at higher elevations ( *Picea abies* , *Pinus cembra* and *Larix decidua* ), while at lower elevations beech, ash and fir forests are more common (*Fagus sylvatica, Fraxinus excelsior, Abies alba*). The area is a popular tourist destination for outdoor recreation, with many tourist facilities, forestry roads, hiking and mountain biking trails and the presence of logging activities (see Salvatori et al. 2024). Within the park, forestry roads are only accessible to vehicles of forestry personnel and residents, including hunters.

Within its boundaries, the park includes state forest reserves where hunting is not allowed, whereas ungulate hunting regularly occurs in the rest of the protected area. The ungulate hunting season in the study area lasts from the first Sunday of September to December 31 of each year, with the exception of Tuesdays and Fridays, and hunting activities are allowed from one hour before sunrise to one hour after sunset, that is, between 05:21 and 18:38 (local solar time in early October). Red deer are usually hunted from vantage points with good visibility, but occasionally can be shot also during walking hunts, with a maximum shot distance allowed of 400 m. Red deer are subject to selective hunting, in which the hunting quota is established based on the census of the previous year, with a balanced proportion of young and adults as well as males and females. During the study period an average of 304 red deer were shot each year in the study area (range: 244–331), indicating a strong hunting pressure. Since 2018, 1 to 3 wolf packs have inhabited the study area, potentially re‐establishing natural predation patterns after centuries of absence. Wolves were strictly protected and thus were not subjected to hunting in the region during the study period.

### 2.2 Data Collection and Management

Between 2020 and 2024, we sampled through systematic camera‐trapping 42 sites along hiking trails and forestry roads. Trail‐based sampling was chosen to increase the detection probability of elusive carnivore species and at the same time to quantify human trail use (Greco et al. 2025). Trails and forestry roads are expected to be the features where human‐related and wolf‐related risks reach their maxima, providing an optimal setting to test the responses of red deer to hunters, outdoor recreation and wolves (Dickie et al. 2017; Greco et al. 2025). Sampling sites were positioned following a regular grid with 2 × 2 km cells within forest environments and excluding areas above the tree line (approximately 1800 m a.s.l.), as close as possible to the centroid of each cell. Camera traps were positioned on trees at about 60 cm above the ground and a distance of 3–5 m from the target trail or forestry road, without the use of bait. Across the 5 years, we maintained the same positions, except for small scale adjustments (e.g., if the tree was cut, a nearby tree was chosen). The sampling took place each year during the ungulate hunting season, and each camera trap remained active for an average of 33 days (SD = 3.87) between early September to mid‐November, for a total of 6883 trapping days across the 5 years. Half of the sampled sites were located within state forest reserves, which do not permit any hunting activity, while the other half were in areas that permit hunting, providing a quasi‐experimental design to test the effect of the late summer–early autumn hunting season on the activity of red deer (Figure 2).

Once the cameras were retrieved, the photographs were processed using the specialised software Wild.AI, hosted on a platform maintained by the University of Florence. This software supports photo storage, organisation, and identification (Niccoli et al. 2025). Image classification was initially performed through the program's built‐in detector, which grouped them into four categories: ‘Blank’, ‘Humans’, ‘Vehicles’ and ‘Wildlife’. Those assigned to the ‘Wildlife’ category were subsequently manually identified to species level. For the purpose of this study, we extracted only data including humans, red deer, and wolves from the full dataset.

### 2.3 Data Analysis

We followed the method outlined by Iannarilli et al. (2025) using hierarchical models paired with cyclic cubic splines to study the activity pattern of the target species in relation to explanatory variables (i.e., predator/prey, outdoor recreation and hunting). This kind of generalised additive mixed models (GAMMs) can estimate animal activity patterns through a periodicity constraint specified by a cyclic cubic smoother of time within the 24‐h cycle (e.g., by hour or half‐hour). Our response variable was the number of events of the target species in each of 48 half‐hourly bins (i.e., intervals of 30 min), per site and year. Given that sunrise and sunset times varied along the study period with shorter day length as the season progressed, we adjusted all timestamps to make them relative to sunrise and sunset times through the *solartime* function from the package activity (Vazquez et al. 2019). This function uses double anchoring to standardise timestamps of animal activity records when day length varies during the study period or across different latitudes. We related red deer activity level and shape to the number of humans (*humans*) and wolf sequences (*wolves*) at each site and to a categorical variable indicating whether each site was open or closed to hunting activities (*hunting*). Human sequences included pedestrians and cyclists. To calculate the continuous independent variables, sequences were defined as observations occurring at intervals of at least 15 min to ensure independence and were corrected by sampling effort subdividing by the number of days each camera was active during each year and multiplying by 100. The number of wolf sequences was corrected for wolf group size to account for the potentially stronger effect of the sequences in which wolves were more numerous (see Ferretti et al. 2023). For example, we considered a sequence with three wolves as equivalent to three sequences with a single wolf. We henceforth refer to this variable as ‘individual wolf events’. To account for random variations across sites and years, we also considered site‐ and year‐random intercepts. For the cyclic cubic splines, we used a number of basis functions *k* = 24, that is, half the number of the half‐hourly bins, placing therefore one knot for every hour. Site‐to‐site variability in activity pattern can be decomposed into the variability in the frequency of site use, or activity level (i.e., how much a site is used), and in the timing of site use (i.e., when a site is used; Figure 1). When analysing animal activity patterns with GAMMs with cyclic cubic splines in relation to spatial covariates, the variability in activity level can be modelled through linear terms that control the overall vertical position of the activity curve on the y axis. The variability in activity timing (i.e., the shape of the activity curve) is instead modelled through smoother terms that are allowed to vary by the spatial covariate of interest (Iannarilli et al. 2025). We tested the potential effect of the three variables on both red deer activity level and timing, including them both as linear and smoother terms, respectively. To test the potential interaction between hunting and outdoor recreation (Prediction P7), we also included a tensor interaction smoother term (*ti*) of time that was allowed to vary by the interaction of *humans* and *hunting*. Numerical covariates were standardised, subtracting the mean and dividing by the standard deviation to make regression coefficients directly comparable and to ease model convergence.

We formulated a full model with the following structure:

$$\begin{array}{c} \mathrm{red}\_\text{deer}\_\text{events}\;\sim \;\text{humans}+\text{hunting}+\text{wolves}\\ \;+s\left(\text{time}\right)+s\left(\text{time}\times \text{humans}\right)+s\left(\text{time}\times \text{hunting}\right)\\ \;+s\left(\text{time}\times \text{wolves}\right)+\mathrm{ti}\left(\text{time}\;\text{×}\;\text{humans}\;\times \text{hunting}\right)\\ \;+1|\text{site}+1|\text{year} \end{array}$$

We compared it with alternative simpler models with no interaction term and with 3, 2, 1 and no covariate, ranking them according to AIC (Burnham and Anderson 2004). When multiple models had a ΔAIC < 2 we model‐averaged their predictions using their AIC weight (Wood 2017).

We followed a similar approach to assess wolf activity patterns, this time including the number of humans (*humans*) and red deer sequences (*red_deer*) as explanatory variables, but not hunting (neither alone nor in interaction), which was highly correlated with the number of red deer sequences (see Results) and was unlikely to directly affect wolves since they were strictly protected during the study period.

$$\begin{array}{c} \text{wolf}\_\text{events}\;\sim \;\text{humans}+\mathrm{red}\_\text{deer}+s\left(\text{time}\right)\\ \;+s\left(\text{time}\;\times \text{humans}\right)+s\left(\text{time}\;\text{×}\;\mathrm{red}\_\text{deer}\right)\\ \;+1|\text{site}+1|\text{year} \end{array}$$

All models were run through the *bam* function of the *mgcv* package (Wood 2011) in the Rstudio environment (RStudio Team 2020), with R version 4.3.1. (R Core Team 2023).

## 3 Results

We collected 18,641 sequences of humans (mean sequences per site and year = 89.62 ± 101.41 SD), 2344 sequences of red deer (11.27 ± 13.50), and 465 individual events of wolves (2.24 ± 4.48; Table S1), across the 5 years and 42 sites. Wolf group size across the years ranged between 1 and 7 individuals (mean = 2.07 ± 1.32).

**FIGURE 3:**
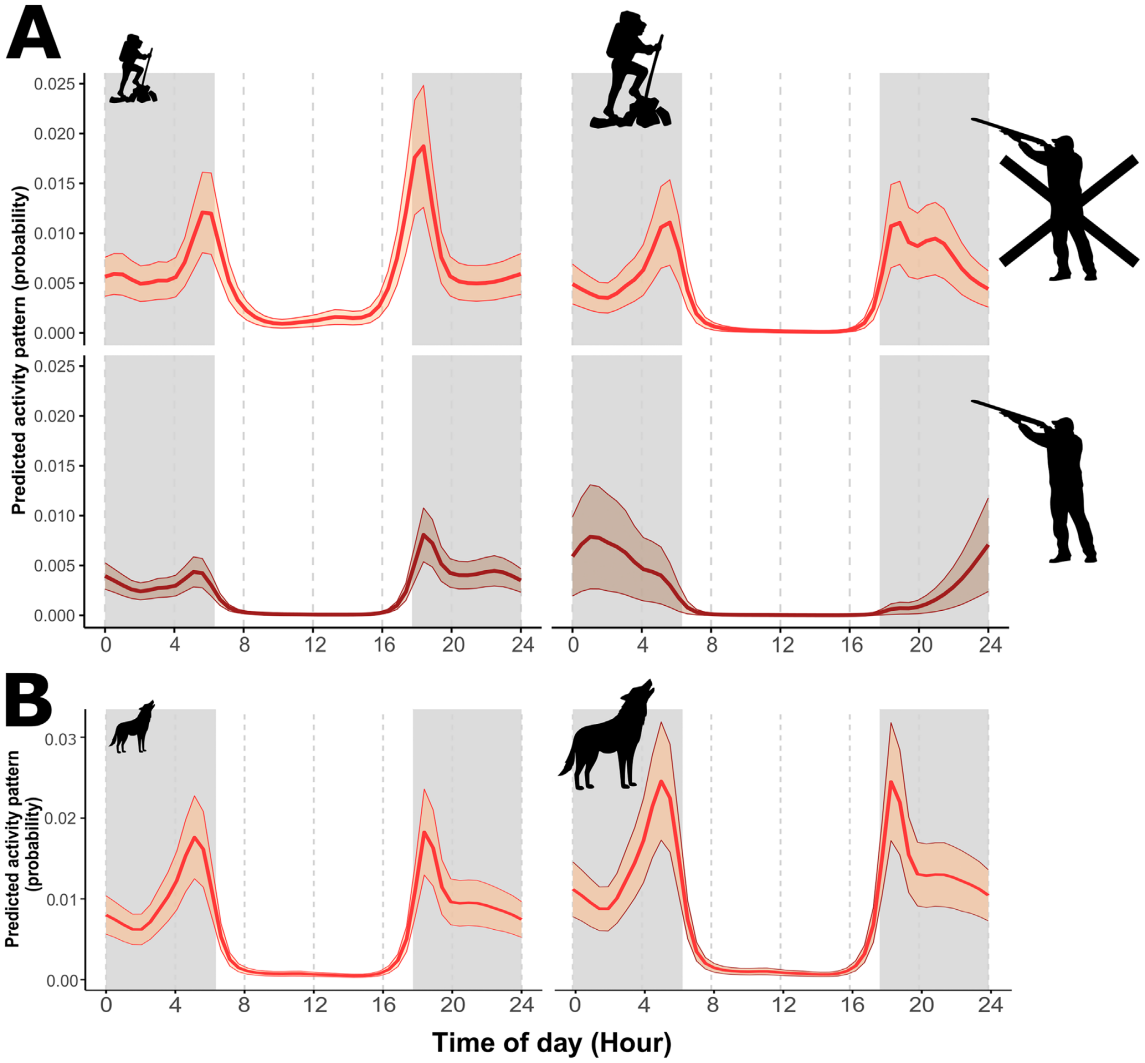
Model predictions of red deer activity patterns in relation to the explanatory variables included in the best GAMM model: the interaction between hunting and outdoor recreation (Panel A), and wolf activity (Panel B). Hunting mediated responses of red deer to outdoor recreation, with stronger effects in presence of hunting. The light grey background indicates nocturnal hours, falling between mean sunset and sunrise time, while ribbons show standard errors around estimates. Low and high levels of the explanatory variables are indicated by the size of the silhouettes, and were defined as the 2.5 and 97.5 percentiles, keeping the other explanatory variable at its mean. Data derive from late summer‐autumn systematic camera‐trapping between 2020 and 2024 in the eastern Italian Alps.

Model selection for red deer indicated the model with *hunting*, *humans* and *wolves*, and the interaction between *hunting* and *humans* as the best one in terms of AIC score, with the second ranked model having a ΔAIC > 2 (Tables 1 and S2). The first ranked model explained 46.7% of the deviance and showed a negative effect of hunting and outdoor recreation on red deer activity level (*βhuntingYES* = −1.41 ± 0.37 SE; *βhumans* = −0.23 ± 0.08; Figure 3A), and a positive effect of individual wolf events (*βwolf* = 0.11 ± 0.03; Figure 3B). Hunting, as well as its interaction with outdoor recreation, had statistically significant effects on the shape of red deer activity, resulting in major changes in the activity curve, whereas wolves had no relevant effect (Figure 3 and Table S2). Hunting strongly decreased diurnal activity and dawn/dusk peaks, and increased the effect of outdoor recreation. In the presence of hunting, high levels of outdoor recreation resulted in a profound modification of red deer activity curve, with a single activity peak around the central hours of the night. In the absence of hunting, high levels of outdoor recreation led to decreased diurnal activity, a lower dusk peak, and higher activity in the first hours of the night, retaining nonetheless a bimodal pattern (Figure 3A).

**TABLE 1:** Model selection table for red deer activity patterns derived from systematic camera‐trapping in the eastern Italian Alps.

Model	Deviance	LogLik	AIC	AIC weight	Delta
Hunting*humans + wolves + time + 1|site +1|year	4966.96	−4156.09	8537.17	1	0
Hunting*humans + time + 1|site +1|year	4986.24	−4165.73	8550.67	0	13.5
Hunting + humans + wolves + time + 1|site +1|year	5148.72	−4246.97	8641.46	0	104.29
Hunting + humans + time + 1|site +1|year	5168.24	−4256.73	8659.03	0	121.86
Hunting + wolves + time + 1|site +1|year	5187.69	−4266.46	8670.49	0	133.32
Hunting + time + 1|site +1|year	5209.71	−4277.47	8684.78	0	147.61
Humans + wolves + time + 1|site +1|year	5255.06	−4300.14	8747.39	0	210.22
Humans + time + 1|site +1|year	5277.84	−4311.53	8757.71	0	220.54
Wolves + time + 1|site +1|year	5286	−4315.61	8758.81	0	221.64
Time + 1|site +1|year	5307.68	−4326.45	8772.74	0	235.57
Time + 1|site	5440.51	−4392.86	8897.69	0	360.52
Time	7440.63	−5392.93	10818.68	0	2281.51

*Note:* Models are ranked based on AIC values.

**FIGURE 4:**
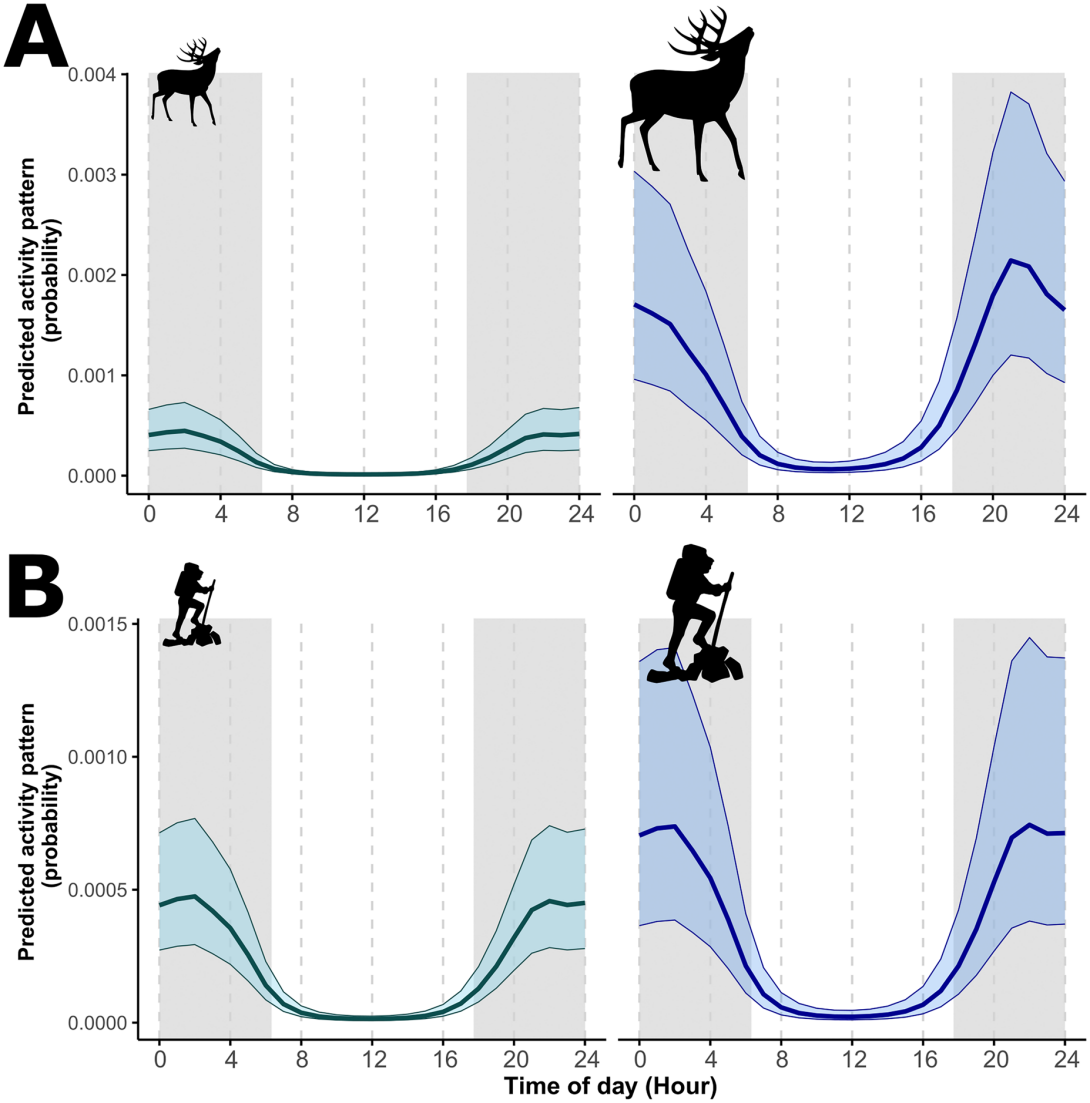
Model‐averaged predictions of wolf activity pattern in relation to red deer (Panel A) and outdoor recreation (Panel B) from the two GAMM models with ΔAIC < 2. Wolves were strongly spatially associated with red deer and remained strictly nocturnal even at low recreation intensity. The light grey background indicates nocturnal hours, falling between mean sunset and sunrise time, while ribbons show standard errors around estimates. Low and high levels of the explanatory variables are indicated by the size of the silhouettes, and were defined as the 2.5 and 97.5 percentiles, keeping the other explanatory variable at its mean. Data derive from late summer‐autumn systematic camera‐trapping between 2020 and 2024 in the eastern Italian Alps.

Model selection for wolf activity patterns identified two models within 2 ΔAIC units: the model with only red deer sequences and the one with red deer sequences and outdoor recreation (Table 2). The first‐ranked model explained 31.5% of the deviance and showed a positive effect of red deer events on wolf activity level (*βred_deer* = 0.33 ± 0.11), but no relevant effect of red deer on the shape of wolf activity, which remained markedly nocturnal (Figure 4A). The second ranked model also reported a positive effect of outdoor recreation on wolf activity level (*βhumans* = 0.31 ± 0.12) but no effect on the activity timing (Figure 4B and Table S3). Since these two models had a ΔAIC < 2, we model averaged predictions using AIC weights to obtain the results shown in Figure 4.

**TABLE 2:** Model selection table for wolf activity patterns derived from systematic camera‐trapping in the eastern Italian Alps.

Model	Deviance	LogLik	AIC	AIC weight	Delta
Red_deer + time + 1|site + 1|year	1438.69	−981.23	2055.65	0.5	0
Red_deer + humans + time + 1|site +1|year	1434.87	−979.31	2055.79	0.47	0.14
Humans + time + 1|site + 1|year	1446.59	−985.17	2062.01	0.02	6.36
Time + 1|site + 1|year	1452.44	−988.1	2064.14	0.01	8.49
Time + 1|site	1466.48	−995.12	2071.12	0	15.47
Time	1837.62	−1180.69	2375.8	0	320.15

*Note:* Models were ranked based on AIC values.

## 4 Discussion

Through a quasi‐experimental design whereby we sampled hunted and nonhunted sites across 5 years, combined with an integrated spatiotemporal statistical approach, we found that hunting was associated with reduced overall activity levels and a marked decrease of diurnality and dawn–dusk peaks in red deer. Human recreational activities were also a relevant predictor of red deer activity levels and timing, with lower overall activity and higher nocturnality at sites with intense outdoor recreation. Crucially, hunting interacted with outdoor recreation exacerbating its impact, with major changes to the red deer activity curve. Meanwhile, wolves did not affect the shape of the red deer activity curve, despite a positive association with red deer activity level. Contrary to our predictions, wolf activity levels were positively and strongly related to red deer site‐use intensity and positively but slightly affected by outdoor recreation. Wolves maintained a strictly nocturnal pattern at both high and low human presence.

Our findings suggest that within human‐dominated landscapes, humans may elicit a greater behavioural response in ungulates than their natural predators—in this case, wolves—both through consumptive and non‐consumptive activities. We found that red deer did not markedly shift their activity peaks in response to wolf presence, rejecting our Prediction P6 (temporal shift in response to wolf). This is in accordance with Vanderlocht, Donini, et al. (2025) that observed a shift towards more diurnal activity by red deer in response to wolf recolonisation only in areas where hunting was not allowed. Indeed, in the presence of recreational hunting the increased diurnality disappeared, indicating that hunting can inhibit temporal antipredator responses. This pattern has also been observed in other European ungulates. For example, Bonnot et al. (2020) showed that in areas across Europe where both hunting and Eurasian lynx ( *Lynx lynx* ) were present, roe deer ( *Capreolus capreolus* ) became increasingly nocturnal, despite marked lynx nocturnality. Similarly, roe deer in the southwestern Alps actively selected areas with high wolf density during hunting periods, indicating a trade‐off between human hunters and natural predators (Ruco and Marucco 2025). These studies suggest that in the presence of hunting, humans are usually perceived by ungulates as a higher risk than natural predators.

The rate at which humans hunt other species is indeed several orders of magnitude higher than natural terrestrial or marine predators, making them exceptionally lethal ‘super‐predators’ (Darimont et al. 2015). For these reasons, when game species encounter human cues, they are more likely to react to avoid immediate mortality, which may explain the spatial and temporal avoidance of primarily lethal humans (hunters), but also nonlethal humans (recreational) (Smith et al. 2024; Trimmer et al. 2017). Indeed we observed that the level of activity of red deer at sites open to hunting was less than half that of sites where hunting did not occur (Prediction P3—lower activity in presence of hunting—supported), with particularly strong reduction of the dawn and dusk activity peaks (Prediction P5—temporal shift in response to hunting—supported), suggesting a strong spatial, and to a lesser extent temporal avoidance. This matches previous evidence on deer proactive avoidance of hunting activities: for example, sika deer ( *Cervus nippon* ) in Japan displayed spatial avoidance of areas with high hunting pressure and were more nocturnal in hunting areas compared to areas where hunting was not permitted (Ikeda and Koizumi 2024). Even though to a minor degree than hunting, outdoor recreation resulted in lower red deer activity, and led to a relatively lower diurnality. These latter results support prediction P1 and P4 (lower activity and temporal shift in response to outdoor activity, respectively) and confirm results from a previous study targeting several areas (Salvatori et al. 2024). Interestingly, the effect of outdoor recreation was mediated by hunting, with stronger alteration of the activity patterns in relation to recreation where hunting was also present, supporting prediction P7 (stronger avoidance of outdoor recreation in presence of hunting). In this case the activity pattern completely shifted from bimodal to unimodal with a single peak during the core hours of the night and no diurnal and crepuscular activity. These results underscore the importance of the exposure to lethal human activities in shaping the responses of wildlife also to nonlethal activities (Zenth et al. 2025). Overall, these human avoidance responses may be effective in reducing mortality, yet they may also entail a substantial physiological cost, altering natural activity patterns, such as foraging, and potentially shaping broader community dynamics (Spitz et al. 2019; Lasky and Bombaci 2023). For example, spatial avoidance of humans may result in clustered hotspots of forest browsing, with alterations of the carbon and nitrogen cycling (Segar et al. 2022; Di Nicola et al. 2023; Donini et al. 2024).

It is important to note, however, that our sampling was concentrated along trails, where human presence is disproportionately high compared to the wider landscape. This may have biased our results by over‐representing the risk of human–wildlife encounters, potentially exaggerating the strength of human effects on red deer activity patterns. Our results should therefore be interpreted relative to trails, given that ungulate responses to human disturbances can vary at different distances from trails, with trail avoidance in high‐disturbance areas (Peters et al. 2025). However, trails are also heavily exploited by wolves for faster and more efficient movements (Dickie et al. 2017; Greco et al. 2025), therefore, they are landscape features where both human‐related and wolf‐related risks should be near their maximum. In addition, Greco et al. (2025) found that the temporal activity curve of red deer exhibited no substantial variation between trail‐based and random‐based sampling, despite a slightly higher nocturnality recorded with the former.

Interestingly, our prediction P2 (lower red deer activity in response to wolves) was rejected, as red deer activity level was positively associated with the site use intensity of wolves. We observed a strong positive relationship between wolf activity rate and red deer site use intensity, suggesting that in our study system, wolves might be favoured in the ‘predator–prey race’, effectively targeting areas and times of day more used by red deer. As highlighted by Smith et al. (2019), the outcome of the behavioural race between prey and predator is highly dependent on which of the two players is more constrained in terms of access to forage and refuge and by predator hunting mode. Cursorial predators that actively chase prey are expected to evoke weaker behavioural responses in prey compared to ambush, sit‐and‐wait predators (Preisser et al. 2007). The landscape of fear shaped by wolves, as cursorial predators and habitat generalists, is indeed highly dynamic (Kohl et al. 2018) and might be significantly less spatially predictable than human‐mediated risk. In our study area, hunting never occurs within state forest reserves, and human disturbance is concentrated along hiking trails and forestry roads, making both lethal and non‐lethal human activities predictable and proactively avoidable compared to natural predators.

In contrast to red deer, wolf activity levels were positively, though slightly, associated with the intensity of site use by humans, rejecting Prediction P8 (lower wolf activity in response to humans). This highlights the plasticity of wolves in adjusting to anthropogenic disturbances, explaining their recent success in recolonising European areas from where they were previously eradicated. However, we observed that wolves remained strictly nocturnal regardless of low or high outdoor recreation, suggesting that wolves ensure stable human avoidance by temporal, rather than spatial segregation (Sunde et al. 2024). Prediction P9—increased wolf nocturnality in response to humans—was therefore rejected. At a broader scale, studies that assessed wolf activity patterns across a gradient of human disturbance have noted that wolves are more diurnal in areas with low or restricted human presence (Ferreiro‐Arias et al. 2024; Mancinelli et al. 2019; Martínez‐Abraín et al. 2023), indicating the pronounced nocturnality we observed as a specific coping mechanism of wolves to human‐dominated landscapes. The observed overlap between red deer and wolf activity patterns therefore is in line with the human super‐predator hypothesis. However, a similar pattern could emerge if wolves do not fear humans directly but instead adjust their activity to track prey that have become more nocturnal in response to human pressure, as may occur where wolves are protected while ungulates are harvested (Kasper et al. 2025). These mechanisms are not mutually exclusive, and could act simultaneously to shape predator–prey interactions.

Overall, our results do not support the human shield hypothesis, as expected in areas where large herbivores are hunted; rather, they show that red deer tend to prioritise the avoidance of lethal and nonlethal human‐driven risks over risk from natural predators. This confirms the conclusions of Gaynor et al. (2025) on HSH being a rare and case‐specific phenomenon, and corroborates findings of Patten et al. (2019) on the increased predator–prey co‐occurrence caused by human avoidance by both actors. The pre‐eminent role of humans in shaping large herbivore behaviour, a signature of the Anthropocene, might therefore undermine the potential for predator‐driven, behaviourally‐mediated trophic cascades, weakening the role of large carnivores as top‐down ecosystem regulators (van Beeck Calkoen et al. 2023; Allen et al. 2017).

The overarching effect of human disturbance can also temporally and spatially ‘squeeze’ prey species, leaving limited opportunity to mitigate risk by natural predators through proactive behavioural adjustments and habitat selection (Crosmary et al. 2012; Vanderlocht, Donini, et al. 2025). As large carnivore populations increase and expand, not just in natural areas but also in human‐dominated landscapes, predator–prey systems must be studied explicitly, including lethal and nonlethal human pressures, to better understand the response of prey species to novel, and often contrasting predatory pressures. Our study focused on a single area but included all combinations of human lethal and nonlethal as well as natural predator threats on red deer, allowing us to disentangle the effects of each factor. Future large‐scale studies may help in assessing whether our results are generalisable to broader spatial contexts, ideally including a gradient of anthropogenic influence and predator presence to better distinguish the respective impacts of humans and natural predators on large herbivores.

## Author Contributions

**Martín Boer‐Cueva:** conceptualization (equal), data curation (equal), formal analysis (equal), software (equal), visualization (equal), writing – original draft (equal), writing – review and editing (equal). **Giulia Bombieri:** conceptualization (equal), data curation (equal), funding acquisition (equal), investigation (equal), methodology (equal), project administration (equal), supervision (equal), writing – original draft (equal), writing – review and editing (equal). **Emma Centomo:** data curation (equal), investigation (equal), writing – review and editing (equal). **Piergiovanni Partel:** conceptualization (equal), funding acquisition (equal), methodology (equal), project administration (equal), resources (equal), supervision (equal), writing – review and editing (equal). **Enrico Dorigatti:** data curation (equal), investigation (equal), writing – review and editing (equal). **Enrico Ferraro:** conceptualization (equal), methodology (equal), writing – review and editing (equal). **Ilaria Greco:** conceptualization (equal), methodology (equal), writing – review and editing (equal). **Francesco Rovero:** conceptualization (equal), funding acquisition (equal), methodology (equal), project administration (equal), resources (equal), supervision (equal), validation (equal), writing – review and editing (equal). **Marco Salvatori:** conceptualization (equal), data curation (equal), formal analysis (equal), investigation (equal), methodology (equal), project administration (equal), software (equal), supervision (equal), validation (equal), visualization (equal), writing – original draft (equal), writing – review and editing (equal).

## Funding

This work was supported by the Wildlife Service of the Autonomous Province of Trento, MUSE ‐ Museo delle Scienze and Parco Naturale Paneveggio Pale di San Martino. M.S. was also funded by Biodiversa+, the European Biodiversity Partnership, in the context of the Big_Picture project under the 2022‐2023 BiodivMon joint call. It is co‐funded by the European Commission (GA No. 101052342) and the Italian Ministry of Universities and Research.

## Conflicts of Interest

The authors declare no conflicts of interest.

## Supporting information


**Table S1:** Summary of sampling effort (camera‐days) and number of sequences of humans, red deer and wolves, for each year. For each category the total number is reported together with the mean and standard deviation (SD) across the 42 sampling sites. The numbers for wolves were corrected for group size, and thus indicate the number of individual wolf sequences.
**Table S2:** Summary output table for the first‐ranked GAMM model for red deer activity.
**Table S3:** Summary output table for the first‐ranked GAMM model for wolf activity.

## Data Availability

The data and R code supporting the findings of this study are provided as Supporting Information.
