# Supplementary material for: Hunting and Outdoor Recreation Affect Large Herbivore Activity Patterns More Than Natural Predators in a Human‐Dominated Landscape
Source: Ecol Evol. 2026 Feb 15;16(2):e73033. doi: 10.1002/ece3.73033 (PMC12906980; doi:10.1002/ece3.73033)
Supplement: Supplementary file 1 — Table S1: Summary of sampling effort (camera‐days) and number of sequences of humans, red deer and wolves, for each year. For each category the total number is reported together with the mean and standard deviation (SD) across the 42 sampling sites. The numbers for wolves were corrected for group size, and thus indicate the number of individual wolf sequences. Table S2: Summary output table for the first‐ranked GAMM model for red deer activity. Table S3: Summary output table for the first‐ranked GAMM model for wolf activity. [file ECE3-16-e73033-s001.docx]

**Appendix**

Table S1. Summary of sampling effort (camera-days) and number of sequences of humans, red deer and wolves, for each year. For each category the total number is reported together with the mean and standard deviation (sd) across the 42 sampling sites. The numbers for wolves were corrected for group size, and thus indicate the number of individual wolf sequences.

| **year** | **effort** | | | **humans** | | | **red deer** | | | **wolves** | | |  |
| --- | --- | --- | --- | --- | --- | --- | --- | --- | --- | --- | --- | --- | --- |
|  | **total** | **mean** | **sd** | **total** | **mean** | **sd** | **total** | **mean** | **sd** | **total** | **mean** | **sd** | |
| 2020 | 1427 | 33.98 | 2.93 | 3655 | 87.02 | 89.83 | 690 | 16.43 | 15.81 | 74 | 1.76 | 3.48 | |
| 2021 | 1394 | 33.19 | 5.51 | 3434 | 81.76 | 86.86 | 438 | 10.43 | 15.24 | 97 | 2.31 | 3.70 | |
| 2022 | 1274 | 30.33 | 3.95 | 3498 | 83.28 | 95.11 | 460 | 10.95 | 13.44 | 86 | 2.05 | 3.79 | |
| 2023 | 1319 | 32.17 | 4.53 | 3264 | 79.61 | 110.70 | 396 | 9.66 | 10.46 | 85 | 2.07 | 3.68 | |
| 2024 | 1469 | 35.83 | 2.43 | 4790 | 116.83 | 121.28 | 360 | 8.78 | 10.79 | 123 | 3.00 | 6.95 | |

Table S2. Summary output table for the first-ranked GAMM model for red deer activity.

| Parametric Coefficients | | | | |
| --- | --- | --- | --- | --- |
|  | **Estimate** | **Standard Error** | **Z value** | **P value** |
| Intercept | -5.72 | 0.29 | -20.24 | <0.001 |
| hunting (Y) | -1.41 | 0.37 | -3.78 | <0.001 |
| humans | -0.23 | 0.08 | -2.98 | <0.01 |
| wolves | 0.11 | 0.03 | 4.26 | <0.001 |
|  |  |  |  |  |
|  |  |  |  |  |
| Approximate significance of smooth terms | | | | |
|  | **Edf** | **Ref.df** | **Chi.sq** | **P value** |
| ti(Time*humans*hunting NO) | 28.21 | 110.00 | 478.10 | <0.001 |
| ti(Time*humans*hunting YES) | 12.06 | 110.00 | 100.28 | <0.01 |
| s(Time*hunting No) | 5.61 | 22.00 | 110.12 | <0.001 |
| s(Time*hunting Yes) | 0.00 | 22.00 | 0.00 | <0.01 |
| s(Time*humans) | 0.00 | 0.00 | 0.00 | 0.99 |
| s(Time*wolves) | 0.45 | 0.79 | 0.15 | 0.70 |
| s(Time) | 14.83 | 22.000 | 340.56 | <0.001 |
| s(Year) | 3.87 | 4.000 | 190.65 | <0.001 |
| s(Site) | 37.94 | 40.000 | 1106.14 | <0.001 |

Table S3. Summary output table for the first-ranked GAMM model for wolf activity.

| Parametric Coefficients | | | | |
| --- | --- | --- | --- | --- |
|  | **Estimate** | **Standard Error** | **Z value** | **P value** |
| Intercept | -8.38 | 0.27 | -30.91 | <0.001 |
| red_deer | 0.33 | 0.11 | 2.96 | 0.003 |
|  |  |  |  |  |
| Approximate significance of smooth terms | | | | |
|  | **Edf** | **Ref.df** | **Chi.sq** | **P value** |
| s(Time*red*_*deer) | 1.22 | 1.87 | 2.46 | 0.29 |
| s(Time) | 5.97 | 22.00 | 138.88 | <0.001 |
| s(Year) | 3.34 | 4.00 | 21.31 | <0.001 |
| s(Site) | 32.31 | 41.00 | 267.33 | <0.001 |
